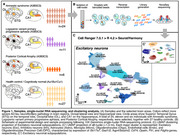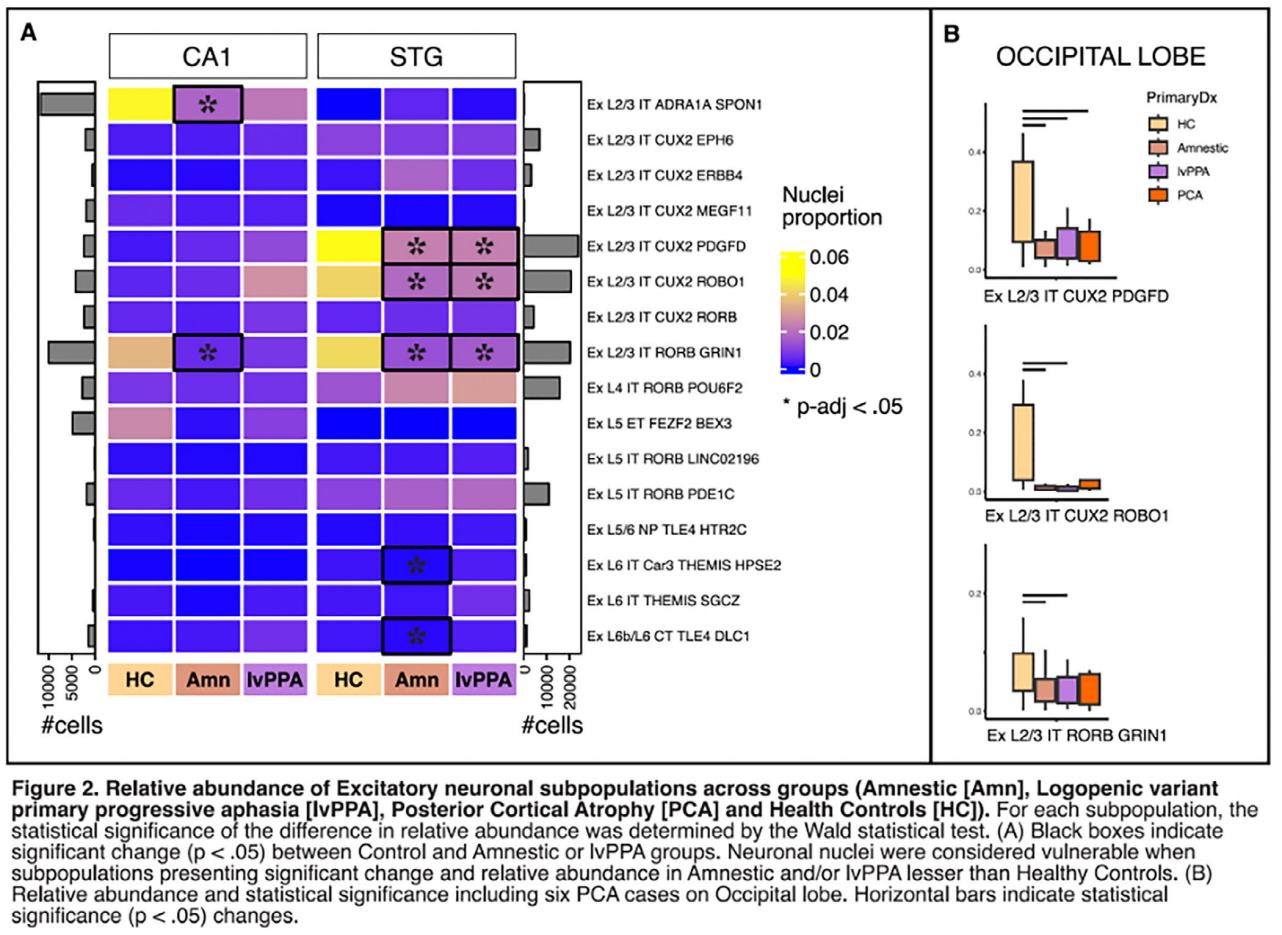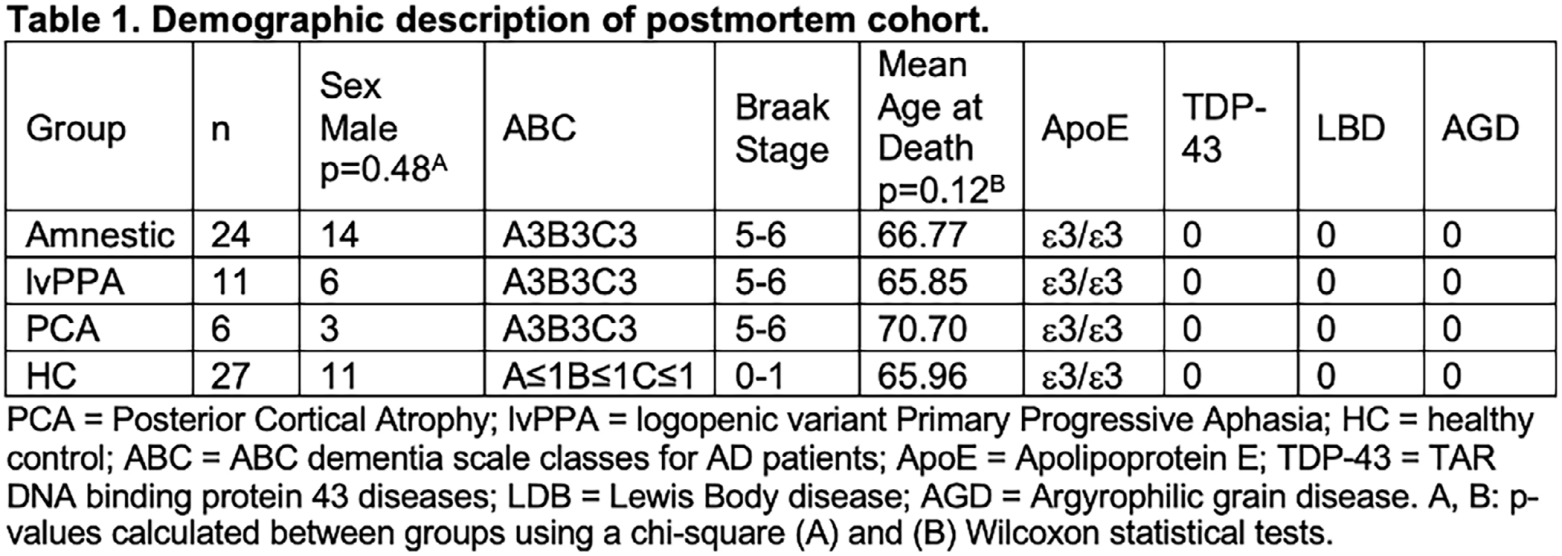# Single‐nuclei transcriptomic identifies neuronal populations vulnerable to AD by leveraging AD variants

**DOI:** 10.1002/alz70855_106654

**Published:** 2025-12-24

**Authors:** Felipe Luiz Pereira, Caroline Lew, Song Hua Li, Liara Rizzi, Igor Prufer Q C Araujo, Alexander V. Soloviev, Salvatore Spina, Jessica E Rexach, William W. Seeley, Claudia Kimie Suemoto, Renata Elaine Paraizo Leite, Kathy L Newell, Bernardino Ghetti, Melissa E. Murray, Lea T. Grinberg

**Affiliations:** ^1^ Memory and Aging Center, UCSF Weill Institute for Neurosciences, University of California, San Francisco, San Francisco, CA, USA; ^2^ Memory and Aging Center, Weill Institute for Neurosciences, University of California San Francisco, San Francisco, CA, USA; ^3^ Department of Human Genetics, David Geffen School of Medicine, University of California Los Angeles, Los Angeles, CA, USA; ^4^ Program in Neurogenetics, Department of Neurology, David Geffen School of Medicine, University of California Los Angeles, Los Angeles, CA, USA; ^5^ Department of Neurology, Memory and Aging Center, University of California San Francisco, San Francisco, CA, USA; ^6^ Physiopathology in Aging Laboratory (LIM‐22), Department of Internal Medicine, University of São Paulo Medical School, São Paulo, São Paulo, Brazil; ^7^ Division of Geriatrics, Department of Internal Medicine, University of São Paulo Medical School, São Paulo, São Paulo, Brazil; ^8^ Department of Pathology, University of São Paulo Medical School, São Paulo, São Paulo, Brazil; ^9^ Indiana University, INDIANAPOLIS, IN, USA; ^10^ Mayo Clinic, Jacksonville, FL, USA; ^11^ Biobank for aging studies of the University of São Paulo, São Paulo, Brazil

## Abstract

**Background:**

Individuals meeting neuropathological criteria for Alzheimer's disease (AD) can exhibit distinct clinical syndromes, suggesting region‐specific neuronal susceptibilities to tau pathology. Amnestic AD cases show greater tau burden in CA1, logopenic variant primary progressive aphasia (lvPPA) in the superior temporal gyrus (STG), and posterior cortical atrophy (PCA) in the occipital cortex. We hypothesize that by contrasting these AD variants and examining their respective pathological hotspots, we can identify the most vulnerable neuronal subpopulations ‐ those that exhibit the greatest reduction in their specific hotspot area compared to other regions. Using single‐nucleus RNA sequencing (snRNA‐seq), we investigate the molecular mechanisms driving this selective neuronal vulnerability.

**Methods:**

We performed snRNA‐seq using the Chromium Single Cell 3′ (10X Genomics, USA) platform on nuclei extracted from the CA1 hippocampal sector, posterior STG, and occipital cortex of postmortem brain tissue from 68 individuals. The cohort included 24 amnestic AD cases, 11 lvPPA cases, and 6 PCA cases (all meeting A3B3C3 pathological criteria), along with 27 healthy controls (A≤1B≤1C≤1). Bioinformatics analysis was conducted using the Seurat library in R. Cell subpopulation comparisons were performed using the Wald statistical test, with *p*‐values < .05 considered significant.

**Results:**

Following quality control, we recovered over 1.58 million nuclei, with an average of 2,129 genes detected per nucleus. UMAP clustering identified 17 inhibitory and 16 excitatory neuronal subpopulations across all three regions. Among these, one excitatory subpopulation was highly enriched in CA1. We identified six excitatory subpopulations (Exc‐subs) exhibiting region‐specific vulnerability. Notably, the Ex L2/3 IT RORB GRIN1 subpopulation showed pronounced vulnerability in CA1 among amnestic cases, in STG among amnestic and lvPPA cases, and displayed a vulnerability trend in the occipital cortex among PCA cases, though not statistically significant.

**Conclusion:**

Our findings suggest that a specific excitatory neuron subpopulation exhibits heightened susceptibility across highly tau‐affected regions in amnestic and atypical AD syndromes, including PCA. Ongoing validation using quantitative pathology aims to confirm these findings, and further case analyses will refine our understanding of the molecular drivers of neuronal vulnerability in AD.